# Use of untreated fresh autologous pericardium for reconstruction of pulmonary valve in tetralogy of Fallot requiring transannular patch: Is it the best material?

**DOI:** 10.1186/1749-8090-10-S1-A197

**Published:** 2015-12-16

**Authors:** Shantanu Pande, Surendra K Agarwal, Aditya Kapoor, Prabhat Tewari

**Affiliations:** 1 Department of Cardiovascular and Thoracic surgery Sanjay Gandhi Postgraduate Institute of Medical Sciences, Lucknow 226014, India; 2 Department of Cardiology Sanjay Gandhi Postgraduate Institute of Medical Sciences, Lucknow 226014, India; 3 Department of Anaesthesia Sanjay Gandhi Postgraduate Institute of Medical Sciences, Lucknow 226014, India

## Background/Introduction

Autologous untreated pericardium is the first material used for reconstruction in cardiac surgery but fell out of repute because of its early failure. Easy availability, and its likeness to valve tissue still upholds the promise of its use as a material for reconstruction of valve.

## Aims/Objectives

To study durability of fresh autologous untreated pericardium (FAP) in reconstruction of a competent pulmonary valve (PV) in TOF requiring transannular patching.

## Method

Between December 2006 and 2012, 98 operated TOF were divided into four groups based on requirement of a monocusp, tricuspid repair of bicuspid PV or need of a tricuspid PV in patients requiring TAP. Group I, monocusp (n = 50), Group II, repaired bicuspid PV valve (n = 6), Group III tricuspid PV (n = 22) and group IV avoiding TAP (n = 20). FAP sutured to the undersurface of TAP and native annulus was used to create a new annulus and a competent PV with one of techniques. Efficiency was assessed by presence of regurgitation or gradient and leaflet thickening and motion by 3 D echocardiography imaging.

## Results

Median age was 11 years (1 - 38), 78 were males. The clinical follow-up is 88% for 57.5 months (33 - 84) while echocardiographic follow-up is 80% for 36 months (6 -72). There was no significant difference in two groups in occurrence of PI (Group I, none 31, mild 12, moderate 6 and severe 1 vs. Group II none 15, mild 5 and moderate to severe 2 vs. Group III, none 4, mild 1 and moderate 1 vs. Group IV, none 16, moderate 2, sever 2, p = 0.59) and RVOT gradient. There was no thickening and calcification in constructed valve.

## Discussion/Conclusion

FAP for reconstruction of pulmonary valve is successful in maintaining functionality, indicated by occurrence of pulmonary insufficiency in reconstructed PV comparable to that of preserved native valve. Further, tricuspid PV has best cooptation amongst groups.

